# Oseltamivir-Resistant Pandemic (H1N1) 2009 Treated with Nebulized Zanamivir

**DOI:** 10.3201/eid1611.100789

**Published:** 2010-11

**Authors:** Liviana Da Dalt, Arianna Calistri, Chiara Chillemi, Riccardo Cusinato, Elisa Franchin, Cristiano Salata, Dino Sgarabotto, Giuseppe Toscano, Antonio Gambino, Giorgio Palù

**Affiliations:** Author affiliations: University of Padova, Padova, Italy (L. Da Dalt, A. Calistri, C. Chillemi, E. Franchin, C. Salata, G. Palù);; Azienda Ospedaliera di Padova, Padova (R. Cusinato, E. Franchin, D. Sgarabotto, G. Toscano, A. Gambino)

**Keywords:** Pandemic (H1N1) 2009, influenza, viruses, antimicrobial resistance, zanamivir, immunocompromised child, letter

**To the Editor:** In late November 2009, a 3-year-old immunocompromised boy experienced an upper respiratory tract infection caused by influenza A pandemic (H1N1) 2009 virus, as demonstrated by a positive result for real-time PCR on a nasal swab specimen. His medical history was notable for a congenital intracardiac tumor; an ABO–incompatible heart transplant at 2 months of age; and an Epstein-Barr virus–related humoral rejection 20 months later that was treated with anti-CD20 and plasmapheresis and continuous immunosuppressive therapy with tacrolimus and everolimus. Thus, a 5-day regimen of oseltamivir treatment was undertaken, and the patient’s clinical signs improved.

However, 3 days after drug treatment was suspended, the child had a relapse and exhibited fever, cough, and mild respiratory distress. The patient had fine crackles in the left posterior basal lung, normal oxygen saturation, and an infiltrate in the left basal lung, observed on chest radiograph. Infection with pandemic (H1N1) 2009 virus was confirmed. He was then transferred to an isolated ward of the pediatric department, and oseltamivir treatment was again initiated and dosages of immunosuppressive drugs were reduced. However, no clinical or virologic responses were observed during the 3 weeks of drug administration.

Over the next month, the oral dosage of oseltamivir was increased twice, without substantial effects on clinical course and viral clearance of the infection (Figure). Because of persistence of infection, the viral neuraminidase gene was sequenced, which showed the H275Y mutation (*1*). We immediately requested zanamivir aqueous solution from GlaxoSmithKline (Brentford, UK), and, after the approval of the hospital’s ethics committee and parents’ consent were obtained, nebulized treatment was carried out for 10 days. Fever and respiratory symptoms and signs resolved after 6 days of treatment and progressive real-time PCR gave negative results. Moreover, at the end of the treatment period, chest radiograph did not show abnormal findings, and results of a hemagglutination-inhibition assay were positive for influenza. No zanamivir-related adverse events were observed, except for a mild bronchospasm that responded to albuterol.

**Figure:**
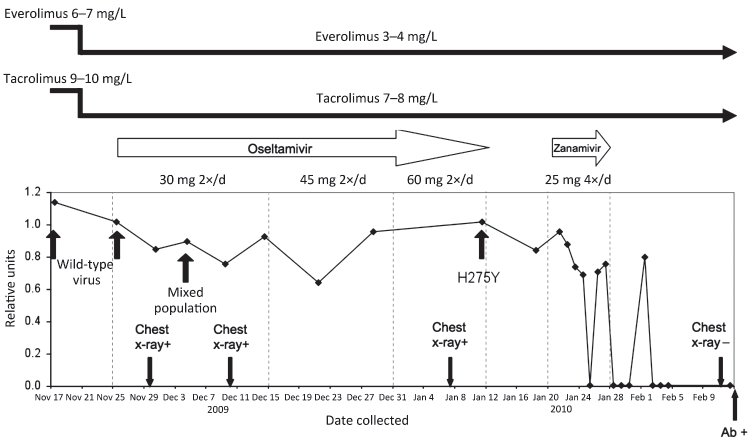
Schematic showing events surrounding oseltamivir-resistant pandemic (H1N1) 2009 virus infection in 3-year-old immunocrompromised child, Italy, in relation to viremia levels, expressed as relative units of influenza A RNA, normalized with respect to the housekeeping gene RNaseP.

Another notable point is that the clinical course of the disease was not severe, although the child was immunocompromised and the infection persisted for almost 2 months. However, influenza virus persistence, possibly caused by inability of the child’s immune system to clear the infection, and prolonged treatment with oseltamivir, led to the appearance of the H275Y mutation. H275Y has been described as the most common mutation that confers oseltamivir resistance in pandemic (H1N1) 2009 infection and has been found in all the resistant isolates reported worldwide (*1*). Consistent with previous reports (*2*), in the patient described here, antiviral drug resistance arose early in the treatment course. Retrospective analysis demonstrated the appearance of a mixed population after ≈2 weeks of drug use with a slow progression toward a pure H275Y variant. This latter finding may be explained by other virologic characteristics of this viral isolate, which is currently undergoing deep sequencing of the full genome.

Zanamivir represents the therapeutic option for patients infected with the H275Y mutation of pandemic (H1N1) 2009 virus. Its licensed formulation as a dry powder is suitable only for patients who can actively use inhaled drugs and thus cannot be used in children <7 years of age (*3*). Intravenous zanamivir solution has been reported to be safe and effective in experimental influenza A virus infection and as compassionate therapy in 2 immunocompromised adult patients who underwent mechanical ventilation for severe pneumonia (*4*,*5*). Moreover, successful use of intravenous zanamivir in a critically ill child, who was immunosuppressed after allogenic stem cell transplantation and infected with oseltamivir-resistant pandemic (H1N1) 2009 virus, has been reported (*6*). In this latter case, the regimen was well tolerated and was associated with a decrease in viral load.

Despite these results showing the efficacy of zanamivir intravenous administration, the inhalatory route for influenza virus–specific drugs should be the first choice, whenever possible, because it delivers therapeutic molecules directly to the site of viral replication, resulting in low systemic exposure. For this reason and because of the mild severity of the patient’s disease, we decided to use zanamivir solution nebulized by aerosol. The compliance to this treatment was easily achieved, and the therapy showed good efficacy and was well tolerated by the child. The minor side effect observed has already been reported in the literature (*7*).

In conclusion, our experience supports the view that in immunocompromised patients with persistent infection, emergence of resistant viral strains should be strictly monitored. In this context, recently developed real-time PCRs for rapid screening of H275Y could be useful (*8*). Moreover, although a direct cause and effect has not been confirmed, this case suggests that aerosolized zanamivir solution can be considered as a therapeutic option in young children with mild respiratory symptoms who are infected with oseltamivir-resistant influenza viruses. Additional studies should be conducted in young patients with more severe disease.
